# The association between signs of medical distress preceding in-hospital cardiac arrest and 30-day survival – A register-based cohort study

**DOI:** 10.1016/j.resplu.2022.100289

**Published:** 2022-08-12

**Authors:** Meena Thuccani, Araz Rawshani, Kristofer Skoglund, Niklas Bergh, Per Nordberg, Malin Albert, Annika Rosengren, Johan Herlitz, Christian Rylander, Peter Lundgren

**Affiliations:** a Department of Molecular and Clinical Medicine, Institute of Medicine, Sahlgrenska Academy, University of Gothenburg, Sweden; b Anesthesiology and Intensive Care, Department of Surgical Sciences, Uppsala University, Uppsala, Sweden; c Prehospen, Centre for Prehospital Research, University of Borås, Sweden; d Region Västra Götaland, Sahlgrenska University Hospital, Department of Cardiology, Gothenburg, Sweden; e Department of Clinical Science and Education, Södersjukhuset, Karolinska Institute, Sweden

**Keywords:** In-hospital cardiac arrest, Outcome, Epidemiology

## Abstract

**Background:**

Identifying signs of medical distress prior to in-hospital cardiac arrest (IHCA) is important to prevent IHCA and improve survival. The primary objective of this study was to investigate the association between signs of medical distress present within 60 minutes prior to cardiac arrest and survival after cardiac arrest.

**Methods:**

The register-based cohort study included adult patients (≥18 years) with IHCA in the Swedish Registry of Cardiopulmonary Resuscitation (SRCR) from 2017-01-01 to 2020–07-15. Signs of distress prior to IHCA were defined as the medical signs arrhythmia, pulmonary oedema, hypotension, hypoxia or seizures present within 60 minutes prior to cardiac arrest (pre-arrest signs). Using multivariable logistic regression, the association between these pre-arrest signs and 30-day survival was analysed in both unadjusted and adjusted models. The covariates used were demographics, comorbidities, characteristics and treatment of cardiac arrest.

**Results:**

In total, 8525 patients were included. After adjusting for covariates, patients with arrhythmia had a 58% higher probability of 30-day survival. The adjusted probability of 30-day survival was 41% and 52% lower for patients with hypotension and hypoxia prior to IHCA, respectively. Pulmonary oedema and seizures were not associated with any change in 30-day survival.

**Conclusions:**

Among signs of medical distress prior to in-hospital cardiac arrest, arrhythmia was associated with a higher 30-day survival. Hypotension and hypoxia were associated with lower survival after IHCA. These findings indicate that future research on survival after cardiac arrest should take pre-arrest signs into account as it impacts the prerequisites for survival.

## Introduction

In Sweden over 2000 in-hospital cardiac arrest (IHCA) occur per year and the prognosis is often poor.^1^ About 80% of patients that gain return of spontaneous circulation (ROSC) after cardiopulmonary resuscitation (CPR) are transferred to the Intensive Care Unit (ICU).^2^ Many of these patients sustain hypoxic organ damage that result in death within days.3., 4. Thus according to the Swedish Registry of Cardiopulmonary Resuscitation (SRCR), in 2019 only approximately 35% of patients survived to 30 days after IHCA.^1^ Identifying the prognostic markers for poor outcome could help develop preventive intervention for cardiac arrest, improve survival when cardiac arrest has occurred, as well as save patients from futile treatment.

Predictors of poor outcome after IHCA include male sex, age 60 or older and several comorbidities.5., 6., 7., 8. The comorbidities associated with poor survival are malignancies, chronic kidney disease, cardiovascular disease and lung disease.6., 7. Intra-arrest factors such as witnessed arrest, shockable first rhythm, early CPR and defibrillation are associated with a better survival outcome.5., 6. Furthermore, longer duration of resuscitation and tracheal intubation seem to be associated with a poor survival outcome.6., 9., 10. Major efforts spent on improving cardiopulmonary resuscitation performance have raised the likelihood of survival but there is still a need for exploring the medical circumstances leading up to the cardiac arrest and its impact on survival.11., 12.

By understanding how the medical circumstances preceding cardiac arrest may affect survival, physicians may be able to prevent cardiac arrest preceded by medical signs of distress (hereinafter referred to as pre-arrest signs). When a cardiac arrest has occurred, the combined knowledge of factors associated with poor outcome including pre-arrest signs, will support the physician in evaluating the decision of discontinuing futile treatment. Therefore, the primary objective of this study was to investigate the association between medical signs of distress present within 60 minutes prior to the cardiac arrest and 30-day survival. The secondary objective was to investigate the association between the pre-arrest signs and shockable first rhythm.

## Methods

### Study design and population

The study design was a population-based retrospective observational study. It is reported according to the STROBE guidelines.^13^ The study population includes all adults (≥18 years) with in-hospital cardiac arrests who received CPR and were registered in the Swedish Registry of Cardiopulmonary Resuscitation (SRCR) between 2017-01-01 and 2020-07-15. The study was approved by the Swedish Ethical Review Authority (DNR 2020-05369). All data was stored and handled in accordance with the European Data Protection Regulation (GDPR).

### Swedish registry of cardiopulmonary resuscitation

The SRCR is a national quality registry started in 1990. Initially only including out-of-hospital cardiac arrests, in 2006 it was expanded to include IHCA. This registry is financed by the Swedish government and counties as well as the Swedish CPR council. In the SRCR, IHCA are defined as cardiac arrests that occur in patients treated in a hospital with an Intensive Care Unit and a resuscitation team. All hospitals in Sweden fulfilling these criteria participate in data registration. Continuous assessments, as part of the registry’s quality work, have demonstrated level of ascertainment exceeding 90% in the last 5 years. Patient data is registered based on Utstein-framework by the nurse/doctor responsible for the patient in the hospital wards.^14^ Additional data on patient background, pre-arrest variables and post-arrest treatment is obtained from the medical records and reported to the registry by a trained nurse.

### Study variables

Relevant data retrieved from the SRCR included demographic, clinical characteristics of the cardiac arrest, pre-arrest signs, date of cardiac arrest and date of death. Demographic data included gender, age at cardiac arrest and comorbidities. Clinical characteristics of the cardiac arrest were variables such as witnessed arrest, telemetry monitoring, first rhythm, CPR before resuscitation team arrived, low-flow time (time from CPR start to ROSC), time from cardiac arrest to ROSC, total no. of defibrillations, if the patient was intubated during the cardiac arrest, if the patient received mechanical chest compressions, if adrenaline was administered and if anti-arrhythmic agents were administered.

The pre-arrest signs were defined as medical signs of distress that were observed within 60 minutes prior to the cardiac arrest. The medical signs that are reported in the present study are listed with definitions according to the SRCR in Table 1. The pre-arrest signs were prospectively recorded from 2018, prior to 2018 the data was reported retrospectively. Thus, data regarding pre-arrest signs from the year 2017 in this study was retrospectively reported.

**Table 1:** Definitions of the medical signs of distress present within 60 minutes prior to cardiac arrest according to the SRCR.

Arrhythmia	Any form of arrhythmia is included
Pulmonary oedema	Symptoms consistent with pulmonary oedema or respiratory distress with lung sounds consistent with pulmonary oedema
Hypotension	Systolic blood pressure < 90 mmHg
Seizures	Any form of seizure
Hypoxia	Blood-oxygen saturation < 90%

The primary outcome was survival to 30 days after the cardiac arrest. The secondary outcome was shockable first rhythm.

### Statistical analysis

Baseline characteristics were presented as number (percentage) for categorical variables and mean (standard deviation) for continuous variables. The baseline characteristics were presented for the overall study population as well as subdivided into patient groups with and without the respective pre-arrest signs and one subgroup with patients without any pre-defined pre-arrest signs.

The association between the pre-arrest signs and 30-day survival outcome was analysed using multivariable logistic regression and presented as odds ratio (OR). As some patients could have more than one pre-arrest sign, the unadjusted odds ratio for each pre-arrest sign was determined using a multivariable logistic regression with all pre-arrest signs. The adjusted models included the following covariates sequentially: demographics (age (as a continuous variable) and sex), comorbidities (previous myocardial infarction (MI), heart failure, diabetes), cardiac arrest clinical characteristics (cardiac aetiology, witnessed arrest, telemetry monitoring, shockable first rhythm), cardiac arrest treatment (adrenaline, anti-arrhythmic agent, defibrillation and were intubated). The covariates selected were variables that presented differently in the patient group with a pre-arrest sign compared to the group without that pre-arrest sign, as seen in the baseline table. Patients with missing data for pre-arrest signs were excluded from the analysis.

The association between the pre-arrest signs and shockable first rhythm was analysed using multivariable logistic regression and presented as crude and adjusted odds ratio. The adjusted model included the same covariates as the logistic regression model for 30-day survival. However, covariates related to cardiac arrest treatment were not adjusted for in the regression model for shockable first rhythm.

All analysis were performed using R 4.0.2 (R Foundation for Statistical Computing, Vienna Austria). Odds ratio from the logistic regressions were presented with 95% confidence interval (CI).

## Results

### Baseline characteristics

Of the 8525 patients included in the study, 38.5% were female, and the average age was 71.9 years (SD 13.4). The most common comorbidity was heart failure. Arrhythmia was the most frequent pre-arrest sign and seizure was the least common pre-arrest sign.

Baseline characteristics for all patients included in the study as well as the subgroups with and without each pre-arrest sign and a separate subgroup without any pre-defined medical signs of distress are presented in Table 2. In Table S1 in the Supplement, baseline characteristics were presented for each pre-arrest sign and subdivided by cardiac and non-cardiac aetiology.

**Table 2:** Baseline characteristics.

	Overall	Arrhythmia		Pulmonary oedema		Hypotension		Hypoxia		Seizure		No pre-defined signs
		Not present	Present	Not present	Present	Not present	Present	Not present	Present	Not present	Present	
n	8525	2905	1668	3188	1151	2912	1188	2707	1374	4392	204	1162
Female (%)	3252 (38.2)	1096 (37.8)	582 (34.9)	1227 (38.6)	399 (34.7)	1123 (38.7)	425 (35.8)	991 (36.7)	533 (39.0)	1657 (37.8)	74 (36.3)	437 (37.7)
Age (mean (SD))	71.9 (13.4)	71.4 (13.6)	72.6 (12.6)	70.6 (14.1)	74.9 (11.4)	71.5 (13.8)	72.0 (12.9)	71.7 (13.7)	72.0 (12.8)	72.1 (13.0)	65.5 (18.1)	70.7 (13.9)
Comorbidities												
Congenital heart defect (%)	24 (0.5)	13 (0.5)	7 (0.7)	14 (0.5)	7 (0.7)	13 (0.5)	9 (0.9)	15 (0.6)	7 (0.6)	22 (0.5)	2 (1.1)	4 (0.4)
Previous MI (%)	1675 (22.5)	560 (19.9)	465 (28.8)	550 (17.8)	377 (34.1)	584 (20.8)	288 (25.0)	612 (23.4)	278 (20.8)	977 (23.1)	32 (16.2)	204 (18.2)
Previous stroke (%)	803 (10.5)	292 (10.2)	171 (10.3)	292 (9.3)	148 (13.0)	277 (9.6)	121 (10.3)	248 (9.3)	161 (11.9)	443 (10.2)	26 (13.1)	83 (7.2)
Heart failure (%)	2346 (32.3)	803 (29.1)	673 (42.3)	455 (14.8)	940 (83.9)	802 (28.8)	431 (38.5)	766 (29.3)	508 (40.2)	1405 (33.7)	48 (24.7)	163 (14.5)
Malignancy (%)	1553 (20.7)	596 (21.1)	257 (15.7)	644 (20.8)	197 (17.7)	554 (19.5)	245 (21.3)	485 (18.4)	299 (22.3)	846 (19.8)	46 (23.2)	232 (20.5)
Diabetes (%)	2137 (27.9)	807 (28.0)	466 (28.1)	813 (25.7)	441 (38.6)	856 (29.6)	326 (27.7)	759 (28.2)	428 (31.3)	1279 (29.4)	50 (24.6)	291 (25.3)
Cardiac arrest characteristics												
Cardiac aetiology (%)	2291 (39.0)	793 (32.1)	701 (60.1)	1020 (37.6)	476 (47.7)	1024 (41.3)	358 (35.5)	1123 (48.3)	286 (23.6)	1549 (41.1)	51 (30.7)	405 (41.9)
Telemetry monitoring (%)	4722 (56.4)	1486 (51.9)	1386 (84.0)	1814 (57.5)	716 (63.3)	1600 (55.6)	806 (69.2)	1681 (62.6)	744 (55.2)	2595 (59.9)	113 (56.2)	610 (52.9)
Witnessed arrest (%)	6888 (81.7)	2353 (81.5)	1530 (92.6)	2632 (83.0)	954 (83.5)	2349 (81.2)	1077 (91.3)	2249 (83.7)	1163 (85.0)	3654 (83.7)	193 (94.6)	911 (79.0)
Shockable first rhythm (%)	1863 (27.5)	1486 (51.9)	690 (47.4)	1814 (57.5)	259 (26.3)	1600 (55.6)	173 (17.9)	1681 (62.6)	119 (10.8)	2595 (59.9)	38 (25.3)	299 (32.1)
Pre-arrest signs												
Arrhythmia (%)	1668 (36.5)	0 (0.0)	1668 (100.0)	636 (22.5)	381 (41.3)	604 (22.7)	363 (39.3)	732 (29.7)	269 (23.1)	1060 (28.0)	45 (26.9)	0 (0)
Pulmonary oedema (%)	1151 (26.5)	542 (19.8)	381 (37.5)	0 (0.0)	1151 (100.0)	525 (19.0)	367 (39.4)	471 (18.4)	462 (39.2)	1018 (25.8)	26 (15.0)	0 (0)
Hypotension (%)	1188 (29.0)	561 (21.5)	363 (37.5)	565 (20.2)	367 (41.1)	0 (0.0)	1188 (100.0)	424 (17.0)	511 (43.3)	987 (26.5)	47 (28.1)	0 (0)
Hypoxia (%)	1374 (33.7)	893 (34.0)	269 (26.9)	717 (25.6)	462 (49.5)	670 (24.5)	511 (54.7)	0 (0.0)	1374 (100.0)	1252 (32.7)	62 (39.5)	0 (0)
Seizure (%)	204 (4.4)	122 (4.3)	45 (4.1)	147 (4.8)	26 (2.5)	120 (4.2)	47 (4.5)	95 (3.6)	62 (4.7)	0 (0.0)	204 (100.0)	0 (0)
Cardiac arrest treatment												
CPR before RRT arrived (%)	6791 (92.6)	2302 (92.4)	1235 (90.9)	2487 (92.1)	925 (91.0)	2344 (92.3)	864 (91.4)	2123 (92.5)	1062 (91.1)	3431 (92.0)	164 (91.1)	920 (93.1)
Intubated during cardiac arrest (%)	4102 (50.6)	1484 (53.7)	587 (37.3)	1477 (48.7)	583 (53.4)	1289 (46.3)	676 (60.5)	1095 (42.5)	834 (63.6)	2083 (50.1)	92 (47.4)	513 (46.3)
Defibrillated (%)	2602 (31.4)	749 (26.4)	782 (48.0)	963 (30.9)	360 (32.3)	934 (32.8)	307 (26.6)	995 (37.6)	268 (19.9)	1383 (32.2)	52 (26.0)	379 (33.3)
No. of defibrillations (mean (SD))	1.4 (2.8)	1.9 (2.6)	1.8 (4.1)	2.0 (3.8)	2.0 (2.8)	2.0 (3.9)	1.7 (2.5)	2.1 (3.9)	2.0 (2.6)	2.1 (3.5)	1.7 (1.5)	1.9 (2.8)
Mechanical chest compression (%)	918 (11.5)	289 (10.6)	147 (9.5)	321 (10.7)	128 (11.9)	257 (9.4)	150 (13.4)	233 (9.2)	163 (12.6)	453 (11.0)	26 (13.5)	110 (10.0)
Adrenaline used (%)	5353 (65.6)	1929 (68.6)	762 (48.4)	1885 (61.4)	799 (72.4)	1667 (59.5)	881 (76.9)	1448 (55.6)	1070 (80.7)	2737 (64.9)	118 (59.9)	653 (57.8)
Anti-arrhythmic used (%)	1208 (15.3)	330 (12.2)	390 (25.2)	439 (14.8)	191 (17.7)	414 (15.1)	166 (15.1)	448 (17.6)	157 (12.3)	658 (16.1)	23 (11.9)	154 (14.1)
Low flow time (min) (mean (SD))	8.9 (16.3)	8.8 (11.9)	6.8 (14.4)	8.2 (13.8)	10.3 (30.5)	8.2 (12.9)	10.8 (31.9)	7.2 (12.7)	12.2 (29.2)	8.9 (19.3)	7.9 (8.1)	7.6 (12.1)
Time from cardiac arrest to ROSC (min) (mean (SD))	9.0 (19.1)	9.3 (21.1)	6.8 (14.3)	8.6 (20.9)	10.2 (29.9)	8.5 (20.7)	10.8 (31.5)	7.1 (12.6)	13.5 (38.6)	9.1 (23.3)	8.1 (8.1)	7.4 (11.9)

Cardiac aetiology is defined as cardiac arrests with presumed cardiac cause. Telemetry monitoring is defined as all patients monitored with telemetry at the time of cardiac arrest. MI = Myocardial infarction, RRT = Rapid response team. Missing data for pre-arrest signs were due to inexplicit reporting of presence or absence of the pre-arrest signs in medical records.

### The association between 30-day survival and pre-arrest signs

**Fig. 1:**
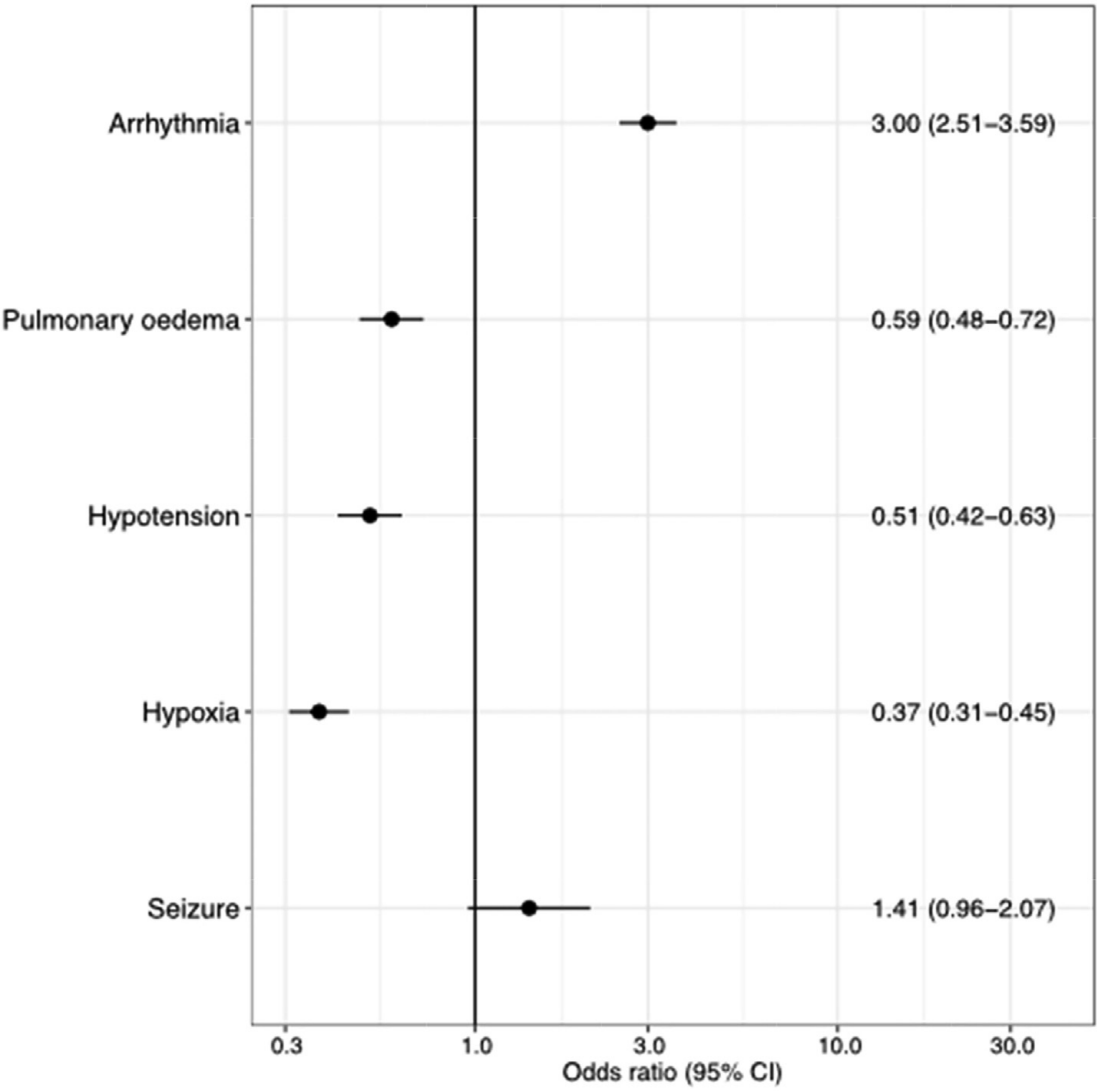
Unadjusted probability (odds ratio) of 30-day survival for each pre-arrest sign. Patients with missing data for pre-arrest signs were excluded from the analysis.

The pre-arrest sign arrhythmia had a higher unadjusted probability of 30-day survival (OR 3.00; 95% CI 2.51–3.59). Pre-arrest signs pulmonary oedema, hypotension and hypoxia had a lower unadjusted probability of 30-day survival (OR < 1), with hypoxia having the lowest probability (OR 0.37; 95% CI 0.31–0.45). The pre-arrest sign seizure was not associated with the probability for 30-day survival, Fig. 1.

**Fig. 2:**
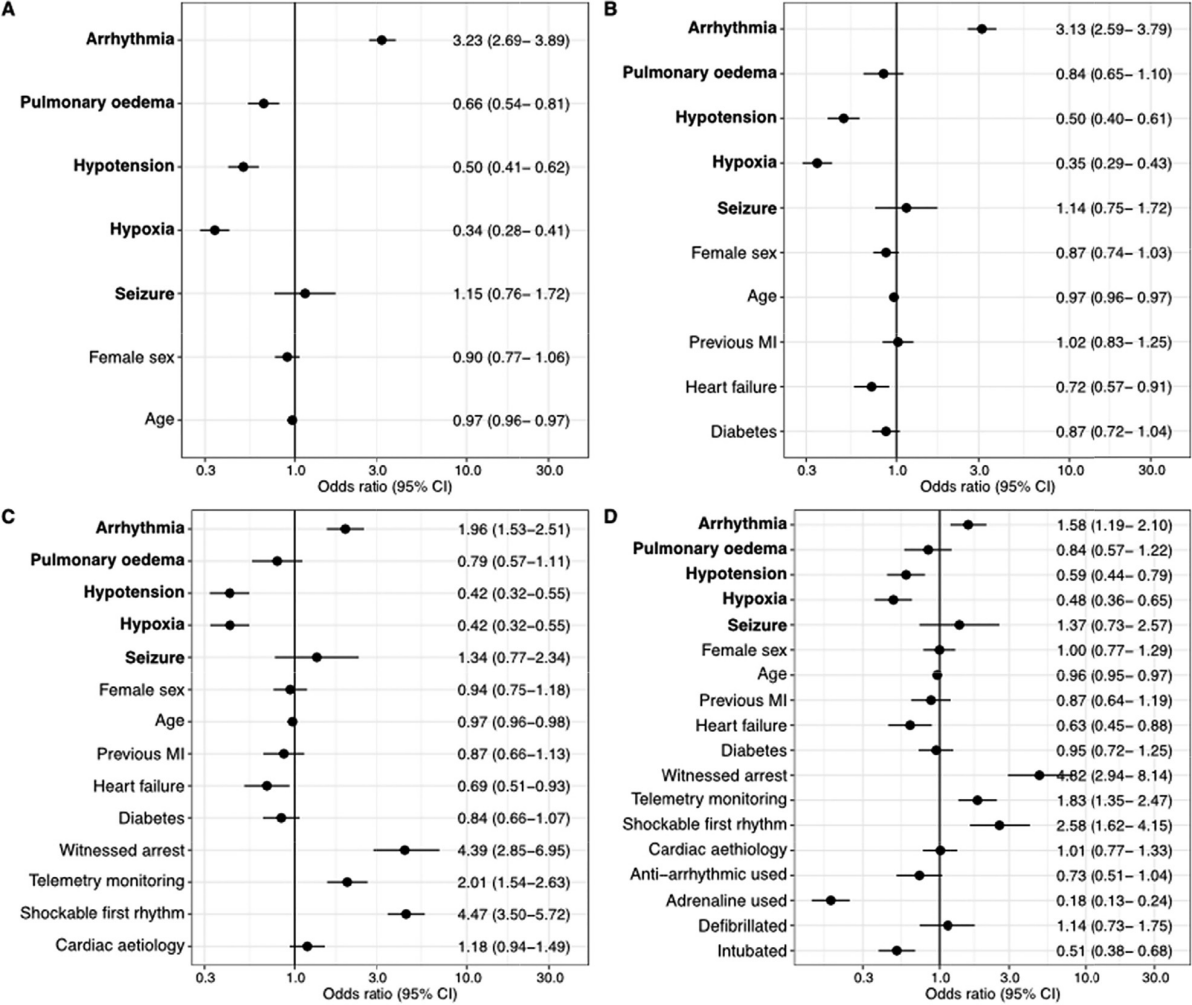
Adjusted probability (odds ratio) of 30-day survival for each pre-arrest sign **A**: model adjusted for sex and age (continuous variable). **B:** model A + comorbidities (previous myocardial infarction (MI), heart failure, diabetes). **C:** model B + arrest characteristics (witnessed arrest, telemetry monitoring, shockable first rhythm and cardiac aetiology). **D:** model C + cardiac arrest treatment (anti-arrhythmic agent used, adrenaline used, defibrillated at any time, intubated). Patients with missing data for pre-arrest signs were excluded from the analysis.

For the pre-arrest sign arrhythmia, sequential adjustment for comorbidities, cardiac arrest characteristics and cardiac arrest treatment attenuated the odds ratio for 30-day survival. Yet, the final adjusted odds ratio still indicated a higher probability for 30-day survival (OR 1.58; 95% CI 1.19–2.10). The association between the pre-arrest signs hypotension and hypoxia and 30-day survival was not affected when adjusting for covariates (OR 0.59; 95% CI 0.44–0.79 and OR 0.48; 95% CI 0.36–0.65, respectively). After adjusting for comorbidities, pulmonary oedema showed no association with 30-day survival, Fig. 2.

### The association between shockable first rhythm and pre-arrest signs

**Fig. 3:**
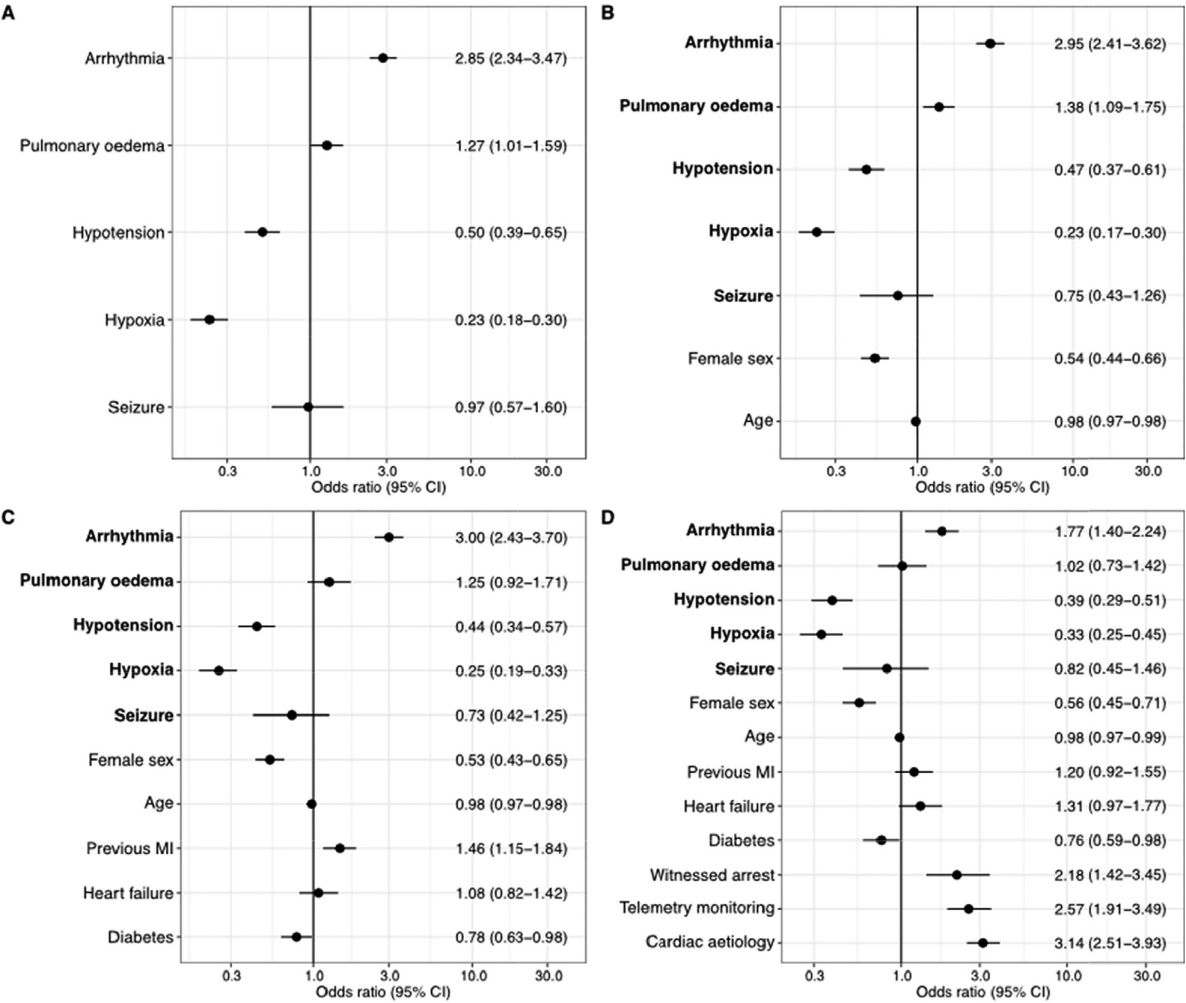
Probability (odds ratio) of shockable first rhythm for each pre-arrest sign **A:** Unadjusted model for probability of shockable first rhythm for each pre-arrest condition. **B:** model A adjusted for sex and age (continuous variable). **C:** model B + comorbidities (previous myocardial infarction (MI), heart failure, diabetes). **D:** model C + arrest characteristics (witnessed arrest, telemetry monitoring and cardiac aetiology). Patients with missing data for pre-arrest signs were excluded from the analysis.

The presence of a shockable first rhythm was more likely for patients with arrhythmia (adjusted OR 1.77; 95% CI 1.40–2.24). After adjusting for comorbidities pulmonary oedema was not associated with shockable first rhythm. Shockable first rhythm was less likely for the pre-arrest signs hypotension and hypoxia in the adjusted models, Fig. 3.

## Discussion

The primary objective of this study was to investigate the association between medical signs of distress presenting within 60 minutes prior to cardiac arrest and 30-day survival. Patients with arrhythmia as a pre-arrest sign was an independent predictor of 30-day survival, as compared to patients without arrhythmia preceding IHCA. Hypotension and hypoxia prior to cardiac arrest were associated with an almost halved adjusted probability for 30-day survival, compared to patients not having these medical signs. Pulmonary oedema preceding cardiac arrest was associated with a lower probability of 30-day survival, however this association was rendered non-significant after adjusting for comorbidities. The pre-arrest sign seizure did not affect 30-day survival.

The results from this study support previous findings that IHCA caused by arrhythmia were associated with increased 30-day survival in patients >70 years.^7^ In contrast, the pre-arrest signs hypotension and hypoxia were associated with a lower probability of 30-day survival. Similarly, in a prospective observational study, hypoxia and hypotension in patients admitted to a general ward were found to predict in-hospital mortality^15^ as well as with the aforementioned study in patients >70 years.^7^

Pulmonary oedema was associated with lower unadjusted probability of 30-day survival, which was rendered non-significant after adjusting for comorbidities. Patients with pulmonary oedema had more comorbidities, previous myocardial infarction, heart failure and diabetes, as compared with patients without pulmonary oedema. A majority of the patients with pulmonary oedema had heart failure. Myocardial infarction and diabetes are often precursor conditions to heart failure.16., 17., 18. Similarly, dyspnoea as a prodromal symptom to cardiac arrest has been shown to have an association with decreased survival,19., 20. which the authors of that study interpreted often as being a symptom of pulmonary oedema or heart failure.^20^ Therefore, it stands to reason that symptomatic heart failure prior to cardiac arrest is a strong prognostic indicator of poor survival.^7^

One of the secondary objectives of this study was to investigate the association between pre-arrest signs and shockable first rhythm. The adjusted probability of shockable first rhythm was 77% higher for patients with arrhythmia. Defibrillation at any time was also more frequent, which suggests that patients with the pre-arrest sign arrhythmia, are more prone to present with or convert to a shockable rhythm during CPR. Atrial fibrillation has been found to be an independent risk factor for ventricular fibrillation.^21^ Additionally, ST segment changes and atrial tachyarrhythmias are common prior to cardiac arrest with cardiac aetiology and are associated with shockable rhythm.^22^ Unfortunately, it is not possible to know which arrhythmias were most prevalent and responsible for the association with shockable first rhythm and survival in the present study.

Shockable first rhythm was 60–70% less likely for patients with hypotension or hypoxia in the adjusted models. Hypotension and acute respiratory insufficiency have been found to be related to PEA/asystole as the first rhythm19., 23. and hypoxia is thought to cause nodal dysfunction with non-shockable rhythm.^22^ Yet, this is not the cause of the lower survival probability observed in this patient group,23., 24. as these pre-arrest signs have an independent association with decreased 30-day survival. This finding suggests that non-shockable rhythm may be a consequence of hypotension and hypoxia prior to cardiac arrest.

Additionally, the longer time intervals to achieve ROSC seen in the patients with hypotension or hypoxia compared to the patients with arrhythmia is possibly representative of a potential to improve advanced life-support treatment. The present study indicates that pre-arrest signs should be given more attention in understanding the underlying pathophysiology resulting in non-shockable rhythm and poor survival. These pre-arrest signs may be helpful in the development of effective treatments in the form of individualized CPR based on these signs and other factors as well. It is also possible that the best treatment for this patient group is established through improving the “Chain of prevention” within the hospital setting.25., 26.

## Strengths and limitations

The association between self-reported prodromal symptoms and survival have previously been investigated.^20^ However, a strength of the present study is that the independent variables investigated are measurable and objective predictors of survival after IHCA.

An additional strength of this study is that the study population was obtained from a comprehensive national registry based in Sweden. Although this could limit the generalisability of the findings, the independent variables were objective and measurable and independent associations with survival were found. Therefore, the findings from this study could be generalised to cardiac arrests with these pre-arrest signs.

A limitation of the current study is the lack of data on the variability within each pre-arrest sign such as the degree of hypoxia or hypotension. Additionally, the pre-arrest sign arrhythmia encompasses many different arrhythmias which have been shown to precede cardiac arrest.^22^

This study only investigated five medical signs prior to cardiac arrest. However, there may be other medicals signs that influence survival or even medical signs that present more than 60 minutes prior to the cardiac arrest, which could be important for improving preventive care for cardiac arrest.

Due to the nature of the retrospective reporting to the registry prior to 2018, a large proportion of patients were excluded from logistic regression analysis due to missing data regarding pre-arrest signs. This missing data is a result of the presence or absence of the pre-arrest sign not being explicitly reported in the medical journals. However, several hundred observations could still be included in the analysis. Hence, the analysis still achieves an acceptable statistical power.

## Conclusion

Among signs of medical distress prior to in-hospital cardiac arrest, arrhythmia was associated with increased 30-day survival. Hypotension and hypoxia were associated with reduced survival after IHCA. These findings warrant that future research on survival after cardiac arrest should take pre-arrest signs into account as it impacts the prerequisites for survival.

## Conflicts of interest

The authors have no conflicts of interest to declare.

## CRediT authorship contribution statement

**Meena Thuccani:** Conceptualization, Methodology, Software, Formal analysis, Investigation, Writing – original draft, Visualization. **Araz Rawshani:** Conceptualization, Methodology, Software, Formal analysis, Investigation, Writing – original draft, Writing – review & editing, Visualization, Resources. **Kristofer Skoglund:** Conceptualization, Methodology, Writing – review & editing, Resources, Visualization. **Niklas Bergh:** Conceptualization, Methodology, Writing – review & editing, Visualization. **Per Nordberg:** Conceptualization, Methodology, Writing – review & editing, Visualization. **Malin Albert:** Conceptualization, Methodology, Writing – review & editing, Visualization. **Annika Rosengren:** Conceptualization, Methodology, Writing – review & editing, Visualization, Resources, Funding acquisition. **Johan Herlitz:** Conceptualization, Methodology, Writing – review & editing, Visualization. **Christian Rylander:** Conceptualization, Methodology, Writing – review & editing, Visualization. **Peter Lundgren:** Conceptualization, Methodology, Investigation, Formal analysis, Writing – original draft, Writing – review & editing, Visualization, Supervision, Funding acquisition.
