# Supplementary material for: The association between signs of medical distress preceding in-hospital cardiac arrest and 30-day survival – A register-based cohort study
Source: Resusc Plus. 2022 Aug 12;11:100289. doi: 10.1016/j.resplu.2022.100289 (PMC9395656; doi:10.1016/j.resplu.2022.100289)
Supplement: Supplementary data 1 [file mmc1.docx]

**Supplement**

**Table S1:** Baseline characteristics stratified by cardiac arrest aetiology

|  | **Overall** | | **Arrhythmia** | | **Pulmonary Oedema** | | **Hypotension** | | **Hypoxia** | | **Seizure** | | **No pre-defined sign** | |
| --- | --- | --- | --- | --- | --- | --- | --- | --- | --- | --- | --- | --- | --- | --- |
| **Aetiology** | **Non-Cardiac** | **Cardiac** | **Non-Cardiac** | **Cardiac** | **Non-Cardiac** | **Cardiac** | **Non-Cardiac** | **Cardiac** | **Non-Cardiac** | **Cardiac** | **Non-Cardiac** | **Cardiac** | **Non-Cardiac** | **Cardiac** |
| **n** | 3590 | 2291 | 466 | 701 | 522 | 476 | 651 | 358 | 928 | 286 | 115 | 51 | 562 | 405 |
| **Female (%)** | 1486 (41.5) | 712 (31.1) | 180 (38.7) | 213 (30.4) | 191 (36.6) | 154 (32.4) | 244 (37.5) | 105 (29.3) | 355 (38.4) | 98 (34.4) | 49 (42.6) | 10 (19.6) | 243 (43.5) | 122 (30.1) |
| **Age (mean (SD))** | 70.8 (14.5) | 72.2 (11.8) | 71.7 (14.1) | 72.2 (12.2) | 74.7 (11.5) | 74.2 (11.1) | 70.3 (14.2) | 73.7 (10.7) | 70.9 (13.7) | 74.2 (10.2) | 63.0 (19.8) | 67.9 (14.3) | 69.8 (15.3) | 70.9 (11.9) |
| **Comorbidities** | |  |  |  |  |  |  |  |  |  |  |  |  |  |
| Congenital heart defect (%) | 16 (0.7) | 6 (0.4) | 3 (0.8) | 3 (0.5) | 4 (0.8) | 2 (0.5) | 8 (1.4) | 1 (0.3) | 5 (0.6) | 1 (0.4) | 1 (0.9) | 0 (0.0) | 2 (0.4) | 2 (0.5) |
| Previous MI (%) | 572 (16.5) | 701 (31.8) | 95 (21.0) | 233 (34.4) | 140 (27.8) | 185 (40.6) | 120 (18.7) | 132 (38.5) | 149 (16.5) | 100 (36.0) | 10 (9.0) | 12 (24.5) | 74 (13.7) | 96 (24.5) |
| Previous stroke (%) | 391 (11.0) | 202 (8.9) | 43 (9.2) | 69 (9.9) | 76 (14.6) | 49 (10.4) | 70 (10.8) | 38 (10.7) | 102 (11.1) | 39 (13.7) | 14 (12.6) | 6 (12.0) | 41 (7.4) | 22 (5.5) |
| Heart failure (%) | 1020 (30.1) | 729 (33.8) | 182 (40.2) | 277 (41.3) | 441 (86.3) | 370 (79.7) | 215 (34.7) | 158 (47.2) | 311 (36.2) | 145 (54.7) | 21 (19.4) | 15 (30.0) | 81 (14.9) | 49 (12.5) |
| Malignancy (%) | 810 (23.3) | 312 (14.0) | 83 (18.1) | 81 (11.8) | 98 (19.3) | 69 (14.9) | 142 (22.3) | 55 (16.0) | 206 (22.7) | 54 (19.4) | 26 (23.0) | 10 (20.8) | 125 (22.8) | 59 (14.8) |
| Diabetes (%) | 943 (26.5) | 676 (29.8) | 132 (28.4) | 210 (30.1) | 196 (37.7) | 185 (39.2) | 177 (27.2) | 107 (30.3) | 273 (29.5) | 106 (37.5) | 19 (16.7) | 17 (33.3) | 138 (24.9) | 88 (21.9) |
| **Pre-arrest signs** | |  |  |  |  |  |  |  |  |  |  |  |  |  |
| Arrhythmia (%) | 466 (21.8) | 701 (46.9) | 466 (100.0) | 701 (100.0) | 129 (29.9) | 221 (55.1) | 151 (28.8) | 186 (58.5) | 128 (16.3) | 110 (44.0) | 26 (26.8) | 17 (41.5) | 0 (0.0) | 0 (0.0) |
| Pulmonary oedema (%) | 522 (23.6) | 476 (31.8) | 129 (34.8) | 221 (38.9) | 522 (100.0) | 476 (100.0) | 170 (31.9) | 172 (54.3) | 270 (33.7) | 159 (62.1) | 13 (13.3) | 8 (18.6) | 0 (0.0) | 0 (0.0) |
| Hypotension (%) | 651 (30.9) | 358 (25.9) | 151 (41.9) | 186 (35.1) | 170 (40.6) | 172 (45.3) | 651 (100.0) | 358 (100.0) | 335 (41.6) | 131 (52.4) | 33 (33.7) | 8 (20.0) | 0 (0.0) | 0 (0.0) |
| Hypoxia (%) | 928 (43.6) | 286 (20.3) | 128 (34.3) | 110 (20.1) | 270 (60.0) | 159 (40.4) | 335 (61.2) | 131 (43.5) | 928 (100.0) | 286 (100.0) | 46 (49.5) | 9 (23.7) | 0 (0.0) | 0 (0.0) |
| Seizure (%) | 115 (4.9) | 51 (3.2) | 26 (6.6) | 17 (2.7) | 13 (2.7) | 8 (1.8) | 33 (5.7) | 8 (2.3) | 46 (5.2) | 9 (3.2) | 115 (100.0) | 51 (100.0) | 0 (0.0) | 0 (0.0) |
| **Arrest characteristics** | |  |  |  |  |  |  |  |  |  |  |  |  |  |
| Cardiac aetiology (%) | 0 (0.0) | 2291 (100.0) | 0 (0.0) | 701 (100.0) | 0 (0.0) | 476 (100.0) | 0 (0.0) | 358 (100.0) | 0 (0.0) | 286 (100.0) | 0 (0.0) | 51 (100.0) | 0 (0.0) | 405 (100.0) |
| Telemetry monitoring (%) | 1697 (48.5) | 1809 (79.5) | 387 (83.8) | 639 (92.1) | 279 (54.5) | 369 (78.5) | 402 (63.1) | 307 (86.5) | 458 (50.3) | 204 (72.6) | 55 (49.1) | 41 (80.4) | 234 (42.2) | 320 (79.2) |
| Witnessed arrest (%) | 2873 (80.8) | 2059 (90.5) | 439 (94.8) | 655 (94.0) | 429 (82.3) | 418 (88.6) | 581 (89.8) | 343 (96.1) | 775 (84.0) | 257 (90.2) | 110 (95.7) | 49 (96.1) | 427 (76.8) | 365 (90.3) |
| CPR before RRT arrived (%) | 2899 (92.6) | 1722 (91.6) | 336 (90.8) | 504 (89.8) | 426 (90.4) | 360 (89.6) | 468 (91.1) | 248 (89.2) | 716 (91.2) | 205 (86.5) | 87 (90.6) | 43 (93.5) | 443 (92.1) | 303 (92.7) |
| Shockable first rhythm (%) | 394 (14.4) | 1014 (49.0) | 143 (35.5) | 355 (56.3) | 83 (19.5) | 157 (35.4) | 64 (12.4) | 96 (30.3) | 51 (7.0) | 59 (23.6) | 11 (13.9) | 25 (54.3) | 65 (15.4) | 221 (59.7) |
| Intubated (%) | 1992 (58.5) | 895 (40.7) | 210 (47.8) | 206 (31.2) | 289 (58.7) | 215 (46.7) | 407 (66.8) | 176 (51.8) | 580 (65.3) | 158 (57.2) | 58 (52.7) | 23 (47.9) | 277 (52.2) | 132 (33.7) |
| Defibrillated (%) | 721 (20.7) | 1173 (52.3) | 176 (38.9) | 384 (56.6) | 133 (26.4) | 191 (41.1) | 137 (21.9) | 136 (38.7) | 146 (16.1) | 90 (31.7) | 16 (14.0) | 30 (61.2) | 108 (19.8) | 240 (59.7) |
| No. of defibrillations (mean (SD)) | 1.0 (2.0) | 2.0 (3.9) | 2.0 (3.4) | 2.5 (5.5) | 1.9 (2.3) | 2.2 (3.3) | 1.6 (2.4) | 2.5 (2.8) | 1.8 (2.2) | 2.4 (3.0) | 1.3 (1.1) | 2.0 (1.8) | 1.5 (1.7) | 2.1 (3.4) |
| Mechanical chest compression (%) | 356 (10.7) | 343 (15.8) | 39 (9.0) | 78 (11.9) | 47 (9.8) | 70 (15.5) | 62 (10.2) | 74 (21.6) | 97 (11.2) | 48 (17.4) | 13 (12.1) | 9 (18.4) | 51 (9.7) | 46 (11.9) |
| Adrenaline used (%) | 2468 (71.8) | 1227 (55.7) | 249 (57.0) | 294 (44.7) | 382 (76.4) | 308 (67.1) | 499 (80.0) | 253 (72.9) | 726 (81.3) | 217 (77.8) | 73 (66.4) | 27 (54.0) | 341 (62.8) | 172 (43.5) |
| Anti-arrhythmic used (%) | 342 (10.4) | 548 (25.2) | 92 (21.5) | 204 (31.2) | 63 (13.1) | 108 (23.8) | 73 (12.3) | 78 (23.1) | 86 (10.0) | 54 (19.9) | 9 (8.4) | 13 (26.5) | 44 (8.5) | 91 (23.3) |
| Low-flow time (mean (SD)) | 10.5 (20.6) | 7.3 (14.3) | 8.2 (16.6) | 6.7 (16.0) | 12.5 (41.8) | 8.5 (16.8) | 13.2 (40.5) | 8.3 (17.6) | 13.7 (34.0) | 7.7 (8.0) | 7.6 (7.4) | 7.7 (8.6) | 8.9 (12.9) | 6.0 (11.5) |
| Time to ROSC (mean (SD)) | 11.1 (25.9) | 7.0 (13.9) | 8.1 (16.2) | 6.3 (15.6) | 12.3 (40.9) | 8.4 (16.6) | 13.2 (40.2) | 8.2 (17.5) | 15.6 (45.5) | 7.7 (7.8) | 7.8 (7.4) | 7.8 (8.5) | 8.7 (12.7) | 5.7 (11.1) |
| **Survival outcome** | |  |  |  |  |  |  |  |  |  |  |  |  |  |
| 30-day survival (%) | 1013 (28.2) | 1078 (47.1) | 228 (48.9) | 410 (58.5) | 117 (22.4) | 153 (32.1) | 146 (22.4) | 108 (30.2) | 185 (19.9) | 54 (18.9) | 51 (44.3) | 26 (51.0) | 216 (38.4) | 251 (62.0) |

RRT = Rapid Response Team

***The survival curve for each pre-arrest sign***


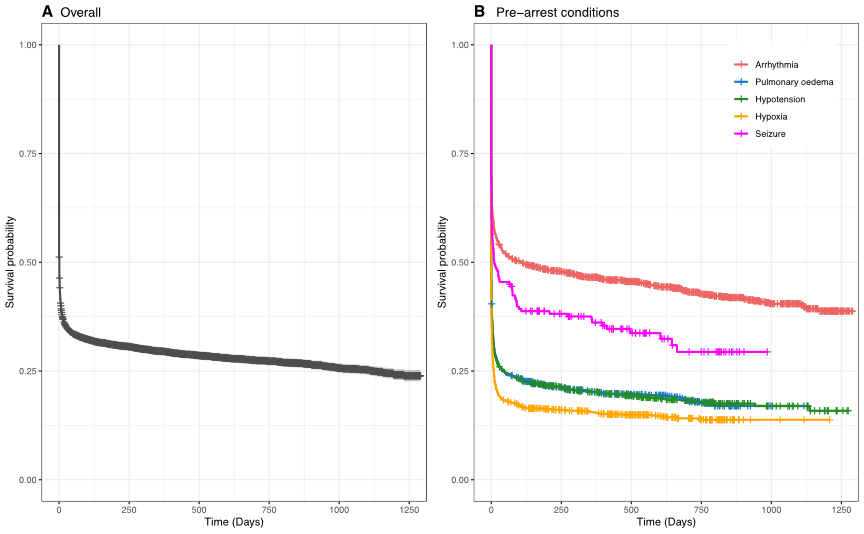
The association between pre-arrest signs and long-term survival were analysed using Kaplan-Meier survival curves for each pre-arrest sign with final follow-up date as 2020-07-15.
All survival curves showed a similar pattern where a majority of lives were lost to the initial cardiac arrest. However, the survival probability was the highest overall amongst patients with arrhythmia prior to the cardiac arrest. Hypoxia was associated with the lowest survival probability overall. Patients with seizures prior to cardiac arrest had also a higher probability of surviving the cardiac arrest, figure S1.

**Figure S1:** Survival curve for each pre-arrest sign

**A**: Kaplan-Meier survival curves from day 0 (cardiac arrest) to last date of follow-up (2020-07-15) for all patients in study population. **B**: Kaplan-Meier survival curves from day 0 (cardiac arrest) to last date of follow-up (2020-07-15) for each pre-arrest sign.

The Kaplan-Meier survival curves show that most patients do not survive the initial cardiac arrest. However, the flattening of the curves indicates that long-term survival becomes more probable for those that survive the initial critical phase. The survival curves also support the results from the logistic regressions that arrhythmia had an overall better survival probability compared to hypoxia and hypotension.

***The association between age and 30-day survival for each pre-arrest sign***

The association between pre-arrest signs and 30-day survival related to age was analysed using a four-knot restricted cubic spline. This analysis was not adjusted for confounders.

A restricted cubic spline of age and 30-day survival for each pre-arrest sign showed that the probability for 30-day survival decreased with increasing age and at 70 and above the OR for 30-day survival decreased to <1 for all pre-arrest signs, figure S2.

**
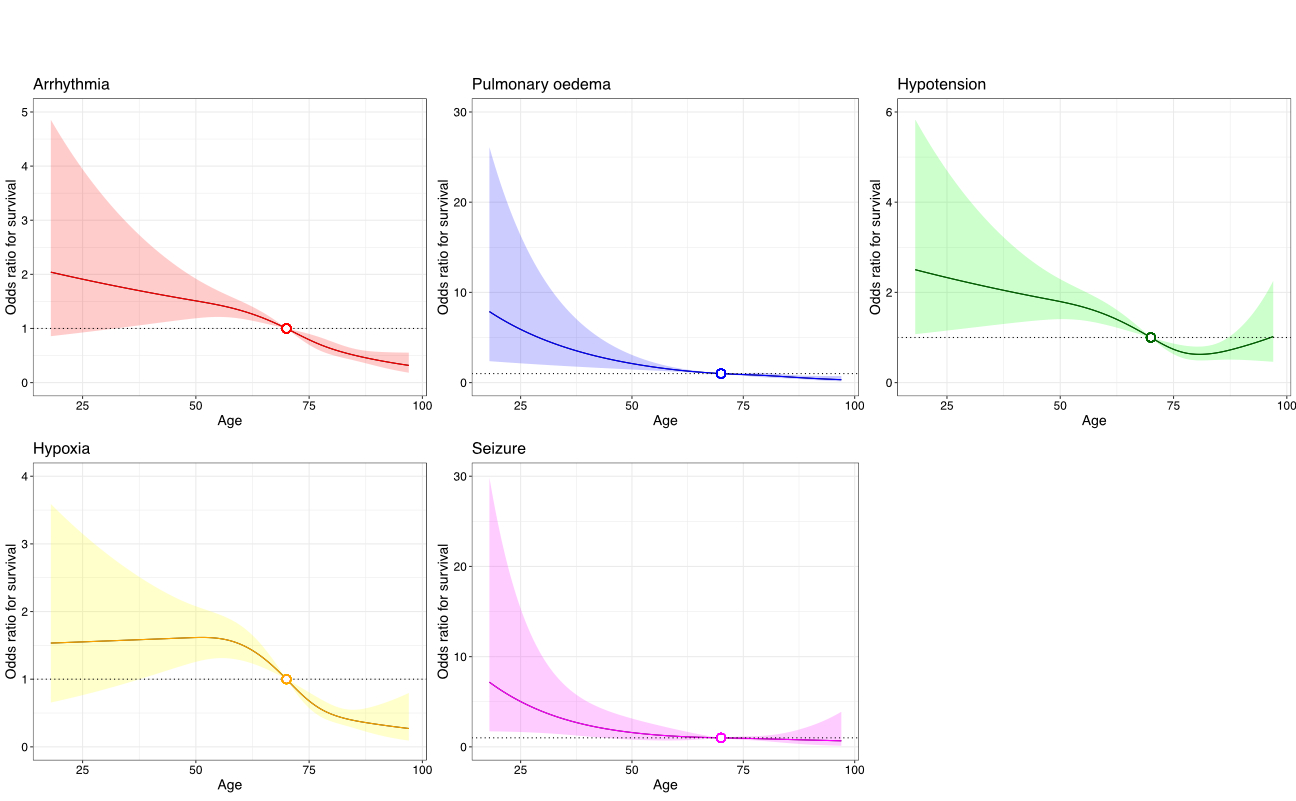
Figure S2:** Probability (odds ratio) of 30-day survival related to age for each pre-arrest sign

Four-knot restricted cubic spline for each pre-arrest sign for probability in odds ratio of 30-day survival related to age in years. Dotted line intercepts with odds ratio 1 on y-axis. The circle on the graph is placed at 70 years of age for all pre-arrest sign.

For each pre-arrest sign the odds ratio for 30-day survival decreases with increasing age and crosses below 1 at the age of 70 years. At 60 years of age the survival probability declines, especially in the hypoxia group. It is possible that with increasing age one becomes more susceptible to hypoperfusion and hypoxia during cardiac arrest, especially when these medical signs proceed the arrest. This could be due to a certain risk profile attributed by comorbidities or an underlying physiological change resulting from aging. Additionally, the increasing mortality could be due to CPR terminated early and Withdrawal of Life-Sustaining Therapy (WLST), which are common medical practices in the older patient population [1]. However, this is rather uncommon for patients at the relatively young age of 60 years. Unfortunately, without adjusting for confounding variables, the possibility of an underlying physiological explanation can only be speculated upon.

**
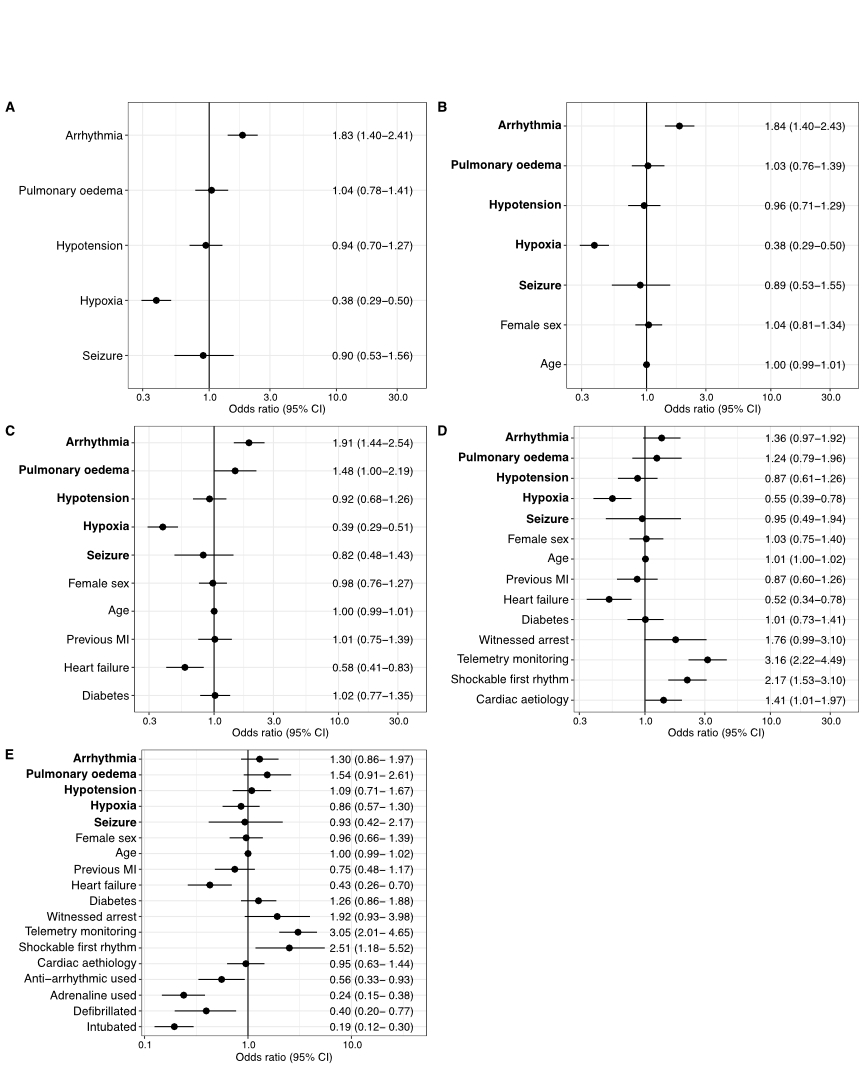
**

**Figure S3:** Probability (odds ratio) of early Return of Spontaneous Circulation (<10 min) for each pre-arrest sign

**A:** Unadjusted model for probability of early Return of Spontaneous Circulation (ROSC) (<10 min) for each pre-arrest sign. **B**: model A adjusted for sex and age (continuous variable). **C:** model B + comorbidities (previous myocardial infarction (MI), heart failure, diabetes). **D:** model C + arrest characteristics (witnessed arrest, telemetry monitoring, shockable first rhythm and cardiac aetiology). **E:** model D + cardiac arrest treatment (anti-arrhythmic agent used, adrenaline used, defibrillated at any time, intubated). Patients with missing data for pre-arrest signs were excluded from the analysis.

**References**

1. Hirlekar, G., et al., *Survival and neurological outcome in the elderly after in-hospital cardiac arrest.* Resuscitation, 2017. **118**: p. 101-106.
